# First In Vivo and Phantom Imaging of Cyclotron-Produced ^133^La as a Theranostic Radionuclide for ^225^Ac and ^135^La

**DOI:** 10.2967/jnumed.121.262459

**Published:** 2022-04

**Authors:** Bryce J.B. Nelson, Simon Ferguson, Melinda Wuest, John Wilson, M. John M. Duke, Susan Richter, Hans Soenke-Jans, Jan D. Andersson, Freimut Juengling, Frank Wuest

**Affiliations:** 1 Department of Oncology, Cross Cancer Institute, University of Alberta, Edmonton, Alberta, Canada;; 2 Cancer Research Institute of Northern Alberta, University of Alberta, Edmonton, Alberta, Canada; and; 3 Edmonton Radiopharmaceutical Center, Cross Cancer Institute, Alberta Health Services, Edmonton, Alberta, Canada

**Keywords:** PET, radiolanthanum, ^225^Ac, theranostics, cyclotron

## Abstract

Theranostic isotope pairs have gained recent clinical interest because they can be labeled to the same tracer and applied for diagnostic and therapeutic purposes. The goals of this study were to investigate cyclotron production of clinically relevant ^133^La activities using natural and isotopically enriched barium target material, compare fundamental PET phantom imaging characteristics of ^133^La with those of common PET radionuclides, and demonstrate in vivo preclinical PET tumor imaging using ^133^La-PSMA-I&T. **Methods:** ^133^La was produced on a 24-MeV cyclotron using an aluminum–indium sealed target with 150–200 mg of isotopically enriched ^135^BaCO_3_, ^nat^BaCO_3_, and ^nat^Ba metal. A synthesis unit performed barium/lanthanum separation. DOTA, PSMA-I&T, and macropa were radiolabeled with ^133^La. Derenzo and National Electrical Manufacturers Association phantom imaging was performed with ^133^La, ^132^La, and ^89^Zr and compared with ^18^F, ^68^Ga, ^44^Sc, and ^64^Cu. In vivo preclinical imaging was performed with ^133^La-PSMA-I&T on LNCaP tumor–bearing mice. **Results:** Proton irradiations for 100 µA·min at 23.3 MeV yielded 214 ± 7 MBq of ^133^La and 28 ± 1 MBq of ^135^La using ^135^BaCO_3_, 59 ± 2 MBq of ^133^La and 35 ± 1 MBq of ^135^La using ^nat^BaCO_3_, and 81 ± 3 MBq of ^133^La and 48 ± 1 MBq of ^135^La using ^nat^Ba metal. At 11.9 MeV, ^135^La yields were 81 ± 2 MBq, 6.8 ± 0.4 MBq, and 9.9 ± 0.5 MBq for ^135^BaCO_3_, ^nat^BaCO_3_, and ^nat^Ba metal. BaCO_3_ target material recovery was 95.4% ± 1.7%. National Electrical Manufacturers Association and Derenzo phantom imaging demonstrated that ^133^La PET spatial resolution and scanner recovery coefficients were superior to those of ^68^Ga and ^132^La and comparable to those of ^89^Zr. The apparent molar activity was 130 ± 15 GBq/µmol with DOTA, 73 ± 18 GBq/µmol with PSMA-I&T, and 206 ± 31 GBq/µmol with macropa. Preclinical PET imaging with ^133^La-PSMA-I&T provided high-resolution tumor visualization with an SUV of 0.97 ± 0.17 at 60 min. **Conclusion:** With high-yield ^133^La cyclotron production, recovery of BaCO_3_ target material, and fundamental imaging characteristics superior to those of ^68^Ga and ^132^La, ^133^La represents a promising radiometal candidate to provide high-resolution PET imaging as a PET/α-therapy theranostic pair with ^225^Ac or as a PET/Auger electron therapy theranostic pair with ^135^La.

## Visual Abstract

**Figure:**
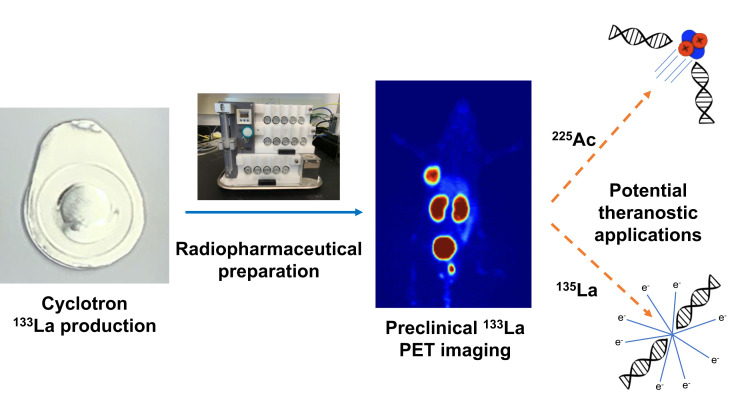

Theranostic pairs in nuclear medicine involve labeling molecular target vectors first with a diagnostic radionuclide, followed by a therapeutic particle–emitting radionuclide (*1*). Both radionuclides should have similar chemical properties, ideally being isotopes of the same element. Theranostics has strong potential in targeted radionuclide therapy, with a diagnostic positron or γ-emitting radionuclide used in PET or SPECT being paired with a therapeutic radionuclide emitting α-particles, β^−^-electrons, or Auger electrons (*2*). Recently introduced ^133^La (half-life [t_½_], 3.9 h), ^132^La (t_½_, 4.8 h), and ^134^Ce (t_½_, 3.2 d)/^134^La (t_½_, 6.5 min) PET radionuclides are uniquely suited as theranostic imaging partners for ^225^Ac (t_½_, 9.9 d) in targeted α-therapy or with ^135^La (t_½_, 19.5 h) in Auger electron therapy (AET) because of their chemical similarity to, and longer half-lives than, the ubiquitous PET radiometal ^68^Ga (t_½_, 68 min) (*2*–*7*). ^225^Ac has shown considerable efficacy in clinical trials for treating metastatic cancers (*2*,*8*). ^132^La has been proposed as a theranostic PET imaging surrogate for ^225^Ac therapy and has displayed in vivo uptake characteristics similar to those of ^225^Ac (*6*). However, there are fundamental imaging limitations inherent in ^132^La because of its high maximum positron emission energy (E_max_) and mean positron emission energy (E_mean_) (E_max_/E_mean_, 3.67/1.29 MeV), which significantly reduces image spatial resolution and contrast compared with other PET radionuclides (e.g., ^18^F E_max_/E_mean_, 0.634/0.250 MeV; ^68^Ga E_max_/E_mean_, 1.90/0.829 MeV; ^64^Cu E_max_/E_mean_, 0.653/0.278 MeV; ^44^Sc E_max_/E_mean_, 1.47/0.632 MeV), and its high-energy and high-intensity γ-emissions, which are problematic from a dosimetric perspective (*3*,*9*). ^133^La has a lower positron emission energy (E_max_/E_mean_, 1.02/0.461 MeV) than ^132^La, ^68^Ga, or ^44^Sc; energy comparable to ^89^Zr (E_max_/E_mean_, 0.902/0.396 MeV), and lower energy and lower-intensity γ-emissions than ^89^Zr, ^44^Sc, or ^132^La (*3*). Here, as outlined in Figure 1, we describe a high-yield cyclotron production method for ^133^La using natural and isotopically enriched ^135^BaCO_3_; phantom measurements comparing fundamental imaging properties of ^133^La with other PET radionuclides, including ^18^F, ^68^Ga, ^64^Cu, ^89^Zr, ^44^Sc, and ^132^La; and the first (to our knowledge) preclinical PET imaging with ^133^La. We have chosen to radiolabel PSMA-I&T for imaging prostate cancers.

**FIGURE 1.**
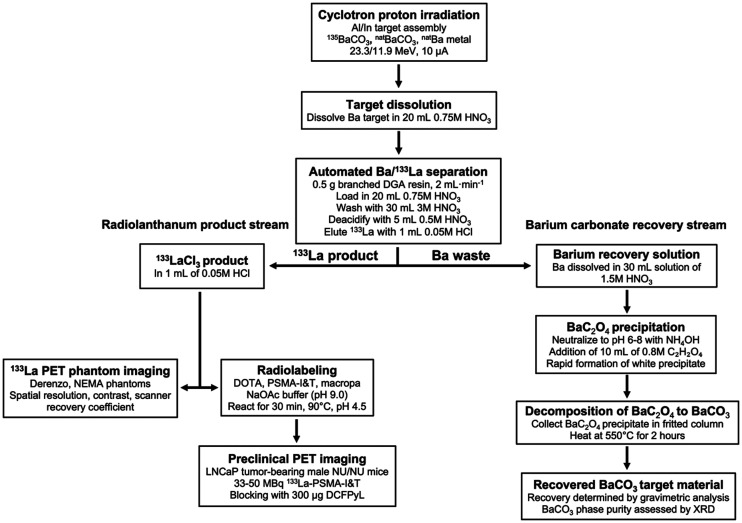
Experimental overview.

## MATERIALS AND METHODS

### Chemicals

Table 1 displays the isotopic compositions of ^135^BaCO_3_, ^nat^BaCO_3_, and ^nat^Ba metal. Isotopically enriched ^135^BaCO_3_ was obtained from Trace Sciences International. Barium carbonate (99.999% trace metals basis), barium metal (99.99% trace metals basis), American Chemical Society reagent–grade concentrated hydrochloric acid (37%), nitric acid (70%), ammonium hydroxide (28%), and periodic table mix inductively coupled plasma optical emission spectrometry (ICP-OES) elemental standards were obtained from Sigma-Aldrich. Oxalic acid dihydrate (99.5%) was purchased from Fisher Scientific. Aluminum disks were obtained from Michaels, and aluminum foil was purchased from Goodfellow Cambridge. Indium wire was purchased from AIM Specialty Materials. Branched diglycolamide resin was purchased from Eichrom. Eckert and Ziegler Isotopes National Institute of Standards and Technology–traceable γ-ray sources were used for high-purity germanium (HPGe) detector energy and efficiency calibration. Thin-layer chromatography silica gel sheets were purchased from Merck. Water (18 MΩ·cm) was obtained from a MilliporeSigma Direct-Q 3 ultraviolet system. ^89^Zr was provided by the Washington University Cyclotron Facility. DOTA was purchased from Macrocyclics. Macropa was purchased from MedChemExpress. PSMA-I&T was obtained from ABX Advanced Biochemical Compounds. DCFPyL was synthesized in-house.

**TABLE 1:** Isotopic Composition of Natural and Isotopically Enriched Barium Target Materials

Target material	^138^Ba	^137^Ba	^136^Ba	^135^Ba	^134^Ba	^132^Ba	^130^Ba
^nat^BaCO_3_/^nat^Ba metal	71.7	11.2	7.9	6.6	2.4	0.1	0.1
^135^BaCO_3_	2.6	0.8	3.6	92.7	0.3	<0.05	<0.05

Data are percentages.

### Instrumentation

Activity and radionuclidic purity were assessed using an Ortec GEM35P4-70-SMP HPGe detector running GammaVision software, with dead times below 25%. Elemental purity was assessed using an Agilent Technologies 720 Series ICP-OES. A NEPTIS Mosaic-LC synthesis unit (Optimized Radiochemical Applications) separated ^133^La from the Ba target solution.

An Eckert and Ziegler AR-2000 radio-thin-layer chromatography imaging scanner quantified the fraction of chelator-bound ^133^La after reaction. Solid targets were manufactured using a Carver model 6318 hydraulic press and an MTI Corp. 10-mm (internal diameter) EQ-Die-10D-B hardened steel die. A Carbolite 16/610-tube 3-zone furnace was used for ^135^BaCO_3_ recovery. X-ray powder diffraction (XRD) patterns were acquired on starting and recovered BaCO_3_ and intermediate BaC_2_O_4_ using a Rigaku Ultima IV x-ray diffractometer to confirm phase identity and purity.

### Cyclotron Targeting and Irradiation

**FIGURE 2.**
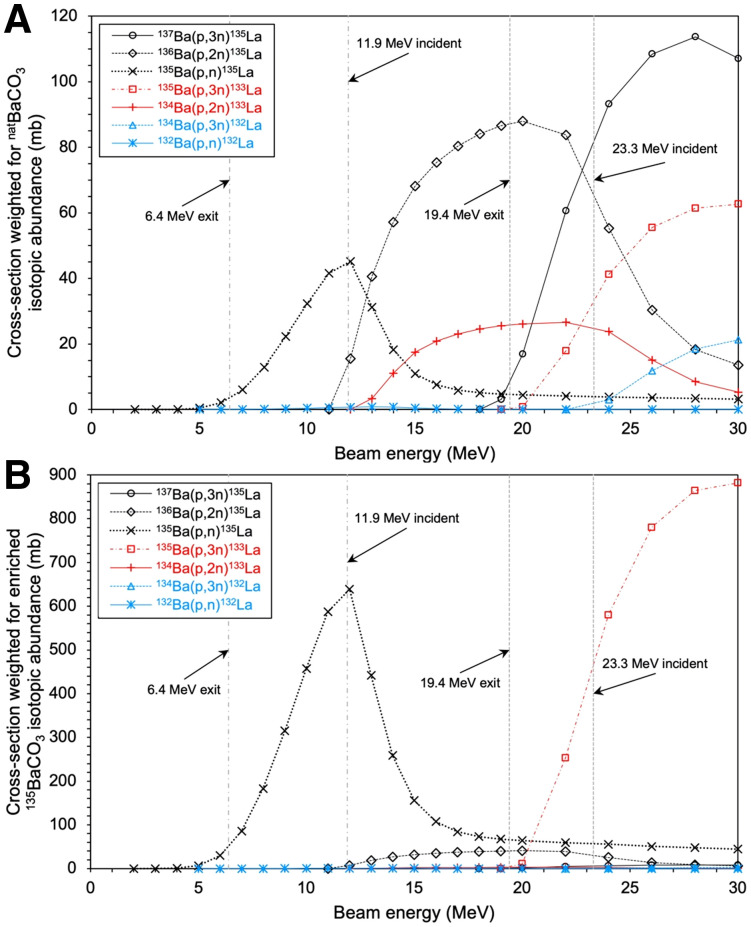
Nuclear reaction cross-section simulation data of proton-induced nuclear reactions on ^132/134/135/136/137^Ba for ^132/133/135^La production weighted for ^nat^Ba isotopic abundance (A) and isotopically enriched ^135^BaCO_3_ abundance (B) (*10*).

Figure 2 depicts nuclear reaction cross-sections for the ^13x^Ba(p,xn)^13x^La reactions of interest for ^132/133/135^La production from the TENDL 2019 library, weighted for ^nat^Ba and isotopically enriched ^135^BaCO_3_ target material (*10*). Cyclotron targets were prepared with 150–200 mg of ^nat^Ba metal, ^nat^BaCO_3_, or enriched ^135^BaCO_3_, a roughened aluminum disk (24 mm in diameter, 1.35 mm thick), indium wire (1 mm in diameter), and aluminum foil (125 µm thick) in a manner similar to that previously described (*3*,*11*). Aluminum was shown to be an adequate substitute for silver, presenting a lower cost and activation. Target components are shown in Supplemental Figure 1 (supplemental materials are available at http://jnm.snmjournals.org). Targets were irradiated for 5–263 min at 11.9 and 23.3 MeV using an Advanced Cyclotron Systems Inc. TR-24 cyclotron, at proton beam currents of 10 µA incident on the target assembly. Higher energy runs (beam-extracted at 24 MeV, 23.3 MeV incident on target pellets, 20.2 MeV exiting Ba metal, and 19.4 MeV exiting BaCO_3_) were performed with 200 mg of barium material with the aluminum target cover facing the beam, to maximize ^133^La production based on TENDL 2019 cross-section simulation data (*10*). During higher-energy runs, a silver disk was placed behind the target to avoid ^13^N production from the ^16^O(p,α)^13^N reaction. For lower-energy runs (18.2-MeV extraction, 11.9 MeV incident on target pellets, 7.8 MeV exiting Ba metal, and 6.4 MeV exiting BaCO_3_), performed to maximize ^135^La production, 150 mg of barium material were used, and the target was installed in reverse with the aluminum disk acting as a degrader to reduce beam energy from 18.2 to 11.9 MeV, as calculated using SRIM (*12*).

### Automated ^133^La Separation and Radiochemical Purity Analysis

^133^La and BaCO_3_ were separated using a process with aspects derived from previous studies (*3*,*4*). The target was opened by peeling back the aluminum cover and placed in a Teflon (DuPont) dissolution vessel. The vessel was filled with 10 mL of 18 MΩ·cm water and sonicated in an ultrasonic bath for 3 min to dislodge the BaCO_3_ from the target backing. Target components were removed and rinsed with 5 mL of 18 MΩ·cm water into the vessel, and 5 mL of 3 M HNO_3_ were added, resulting in a 0.75 M HNO_3_ reaction mixture that dissolved the BaCO_3_ in 5 min. This solution was passed through a solid-phase extraction cartridge containing 0.50 g of branched diglycolamide resin (conditioned with 10 mL of 3 M HNO_3_) and washed with 50 mL of 3 M HNO_3_ to remove residual barium and other metal impurities, followed by column deacidification with 5 mL of 0.5 M HNO_3_. Flow rates were kept below 2 mL·min^−1^ to avoid ^133^La loss from the resin. ^133^LaCl_3_ was eluted using 1 mL of 0.05 M HCl. After passing through the resin, the first 30 mL of process solution were diverted to a collection vial for subsequent BaCO_3_ recovery. After separation, target components were sonicated in 18 MΩ·cm water for reuse. Radionuclidic and elemental purity of ^133^LaCl_3_ was determined by HPGe γ-ray spectroscopy and ICP-OES.

### BaCO_3_ Target Material Recovery

The 30 mL of barium recovery solution were neutralized to pH 6–8 with NH_4_OH. Ten milliliters of 0.8 M C_2_H_2_O_4_ were added to the recovery solution to precipitate BaC_2_O_4_. The solution was passed through a fritted column to trap BaC_2_O_4_ and washed with 50 mL of 18 MΩ·cm water. BaC_2_O_4_ was removed from the column and then heated to 550°C for 2 h in a sealed tube furnace with an airflow of 20 mL/min to decompose BaC_2_O_4_ to BaCO_3_ while avoiding conversion to BaO (*13*). Waste gases from decomposition were vented to a fume hood. Recovery was quantified by gravimetric analysis of dried samples and tracked by HPGe γ-spectroscopy using γ-emissions from ^135m^Ba (268 keV; t_½_, 28.7 h). Samples of purchased BaCO_3_, precipitated BaC_2_O_4_, and recovered BaCO_3_ were analyzed by XRD to identify the product and evaluate its quality.

### Phantom Imaging

Phantom imaging was performed using Derenzo and National Electrical Manufacturers Association (NEMA) image-quality phantoms on an Inveon PET/CT scanner (Siemens Preclinical Solutions), as described by Ferguson et al. (*14*). The Derenzo phantom, used to investigate image contrast and spatial resolution, consists of sections with rods of varying diameters (0.8, 1.0, 1.25, 1.5, 2.0, and 2.5 mm) that are filled with the radionuclide of interest diluted in 20–30 mL of water. The NEMA phantom, used to investigate image noise, spillover ratio, and recovery coefficient, consists of several fillable sections including two 7.5-mm-diameter cold-air and water cylindric volumes. NEMA and Derenzo phantom scans for ^133^La, ^132^La, and ^89^Zr were acquired in list mode, binned into sinograms, and reconstructed with the default filtered backprojection, ordered-subset expectation maximization, and maximum a posteriori estimation algorithms. Acquisition, data processing, and evaluation followed the same procedure as used by Ferguson et al. (*14*) for ^18^F, ^64^Cu, ^68^Ga, and ^44^Sc to enable direct comparison of the different radionuclides’ imaging performance.

### Radiolabeling of DOTA, PSMA-I&T, and Macropa with ^133^La

Similar to techniques in previous studies (*3*,*4*), the activity of a 500-µL ^133^LaCl_3_ aliquot was measured, and the solution pH was adjusted to 4.5 with 50 µL of NaOAc buffer (pH 9.0). A 100-µL volume of this ^133^La solution (5–150 MBq) was reacted with 0.1–20 µg of DOTA, PSMA-I&T, and macropa dissolved in 50 µL of 18 MΩ·cm water at 90°C for 30 min. Each solution was analyzed using radio–thin-layer chromatography on silica plates to determine radiochemical purity and incorporation with 0.1 M citric acid buffer as the mobile phase.

### Preclinical PET Imaging

Animal studies using LNCaP tumor–bearing male nu/nu nude mice (Charles River Laboratories) were performed according to the guidelines of the Canadian Council on Animal Care and approved by the local Cross Cancer Institute Animal Care Committee. Static PET image scans (20-min duration) of ^133^La-PSMA-I&T at 60 min after injection were performed on an Inveon PET/CT scanner (Siemens Preclinical Solutions). Blocking experiments were performed using the PSMA-targeting agent DCFPyL. Radiotracer (33–50 MBq of ^133^La-PSMA-I&T in 80–120 µL of NaOAc/saline) and blocking compound (300 µg of DCFPyL, dosed 5 min beforehand) were injected into the tail vein of isoflurane-anesthetized mice (100% oxygen; gas flow, 1.5 L/min), the mice were placed in a prone position into the center of the field of view, and body temperature was kept constant at 37°C. A transmission scan for attenuation correction was not acquired. The frames were reconstructed using ordered-subset expectation maximization and maximum a posteriori algorithms. No correction for partial-volume effects was applied. The image files were processed using ROVER software (version 2.0.51; ABX GmbH).

### Statistical Analysis

All data are given as mean ± SD (*n* ≥ 3).

## RESULTS

### Cyclotron Targeting and Irradiation

Average end-of-bombardment activities (*n* = 3) of ^133^La and coproduced ^135^La for 100 µA·min runs (10 µA for 10 min) at 11.9- and 23.3-MeV beam energies with different barium target materials are summarized in Table 2. Irradiating enriched ^135^BaCO_3_ at 23.3 MeV resulted in a significant increase in ^133^La production compared with ^nat^BaCO_3_ and ^nat^Ba metal. Irradiating recovered ^nat^BaCO_3_ at 23.3 MeV for 100 µA·min yielded 57 ± 1 MBq of ^133^La and 36 ± 1 MBq of ^135^La, similar to yields for fresh ^nat^BaCO_3_.

**TABLE 2:** Average Experimental (*n* = 3) End-of-Bombardment Activities (MBq) and Saturated Yields (MBq/µA) of ^133/135^La for 100-µA·Min Runs at 11.9- and 23.3-MeV Incident Energies for Different Barium Target Materials

Beam energy (MeV)	^135^BaCO_3_ target yields		^nat^BaCO_3_ target yields		^nat^Ba metal target yields	
	^135^La	^133^La	^135^La	^133^La	^135^La	^133^La
11.9	81 ± 2 (79); y = 1,377 ± 31	0	6.8 ± 0.4 (5.9); y = 115 ± 6	0	9.9 ± 0.5 (10); y = 167 ± 8	0
23.3	28 ± 1 (31); y = 475 ± 11	214 ± 7 (279); y = 736 ± 25	35 ± 1 (41); y = 598 ± 9	59 ± 2 (61); y = 204 ± 8	48 ± 1 (61); y = 809 ± 17	81 ± 3 (94); y = 277 ± 9

y = saturated yield in MBq/µA. Theoretic end-of-bombardment activities calculated with TENDL are in parentheses.

### ^133^La Separation and Radiochemical Purity Analysis

Table 3 contains ICP-OES elemental purity results for the ^133^LaCl_3_ product. After removal from the reactor after sonification, the aluminum target backing and cover contained no detectable ^133^La activity. The entire separation took approximately 50 min. Over 92% of decay-corrected ^133^La was recovered in 1 mL of 0.05 M HCl, and HPGe analysis of the ^133^LaCl_3_ product produced with ^nat^BaCO_3_ showed small activities of ^131^La (t_½_, 59 min) and ^132^La (t_½_, 4.8 h) with no other observed radionuclidic impurities, similar to previous findings (*3*). ^131^La and ^132^La were not observed in ^133^LaCl_3_ produced with isotopically enriched ^135^BaCO_3_. Elemental purity determined by ICP-OES of ^133^LaCl_3_ produced with fresh and recovered BaCO_3_ target material was superior to ^133^LaCl_3_ previously produced with barium metal as described in a previous publication (*3*).

**TABLE 3:** ICP-OES Analysis (*n* = 3) of ^133^LaCl_3_ Produced with Different Barium Target Materials

	Elemental concentration (ppb)		
Metal	Fresh BaCO_3_	Recovered BaCO_3_	Barium metal
Zinc	7.4 ± 1.7	5.5 ± 2.4	76 ± 55
Iron	3.2 ± 0.4	2.1 ± 0.8	16.8 ± 11.7
Aluminum	18 ± 2	16 ± 1	37 ± 19
Barium	240 ± 179	128 ± 108	1,150 ± 360
Indium	2.5 ± 1.2	3.9 ± 1.5	3.1 ± 0.9
Copper	5.5 ± 0.3	5.3 ± 0.1	5.3 ± 0.4

Data for barium metal are from Nelson et al. (*3*).

### Enriched ^135^BaCO_3_ Recovery

**FIGURE 3.**
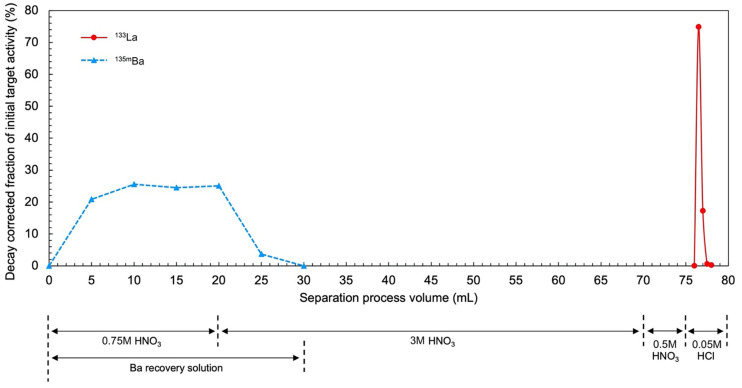
Decay-corrected fraction of initial ^135m^Ba and ^135^La target activity in solid-phase extraction cartridge eluate as function of process volume.

Figure 3 depicts the decay-corrected fraction of total ^135m^Ba and ^135^La activity as a function of process volume. The solution was collected in fractions (5 mL for 0–75 mL, 0.5 mL for 75–80 mL) after flowing through the resin, and each fraction was analyzed on the HPGe to quantify ^135m^Ba and ^135^La activity via their respective 268- and 481-keV γ-emissions. Over 99.7% of decay-corrected ^135m^Ba activity was recovered in the first 6 fractions, with no detectable contributions from additional fractions; therefore, only the first 30 mL of process solution were collected for recovery.

BaC_2_O_4_ formed a white precipitate and was collected by the fritted column. After BaC_2_O_4_ thermal decomposition to BaCO_3_ from heating at 550°C, gravimetric analysis indicated a recovery of 191.1 ± 3.2 mg, which for a 200.3 ± 0.3 mg initial target pellet mass corresponds to a BaCO_3_ recovery of 95.4% ± 1.7% (*n* = 3).

**FIGURE 4.**
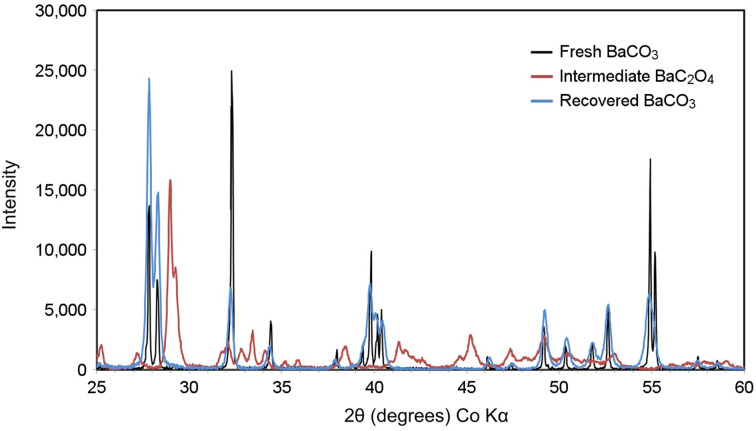
Background-stripped XRD diffractograms of fresh BaCO_3_, intermediate BaC_2_O_4_, and recovered BaCO_3_.

Figure 4 depicts the XRD diffractograms acquired for fresh BaCO_3_, intermediate BaC_2_O_4_, and recovered BaCO_3_ material. Complete XRD diffractogram data are in Supplemental Tables 1–3 and Supplemental Figures 2–4. The absence of unexplained reflections in all 3 patterns, compared with standard reference lines, confirmed the high phase purity of the compounds and the complete conversion of BaC_2_O_4_ to BaCO_3_ (*15*).

### Phantom Imaging

**FIGURE 5.**
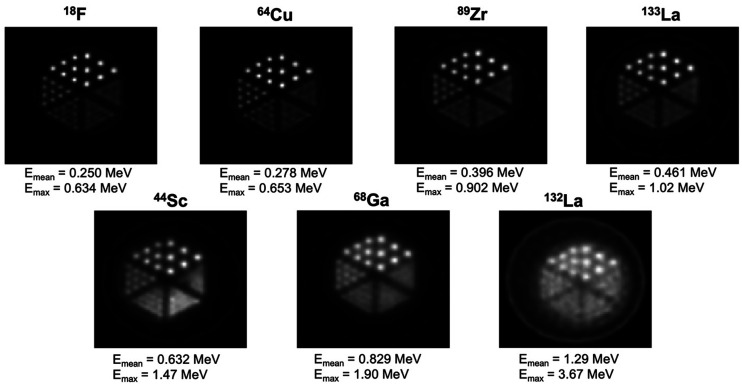
Derenzo phantom images reconstructed with maximum a posteriori estimation for different PET radionuclides, presented in order of increasing positron emission energy. ^18^F, ^64^Cu, ^44^Sc, and ^68^Ga data were taken from Ferguson et al. (*14*).

Figure 5 depicts Derenzo phantom scans with the mean and maximum positron energies of ^133^La, ^132^La, and other commonly used PET radionuclides. Derenzo phantom scans acquired with ^18^F, ^64^Cu, ^89^Zr, ^133^La, ^44^Sc, ^68^Ga, and ^132^La clearly show that lower mean and maximum positron energies improve PET image spatial resolution and contrast. ^133^La exhibits spatial resolution similar to that of ^89^Zr, is an improvement over ^44^Sc and ^68^Ga, and is superior to ^132^La.

**FIGURE 6.**
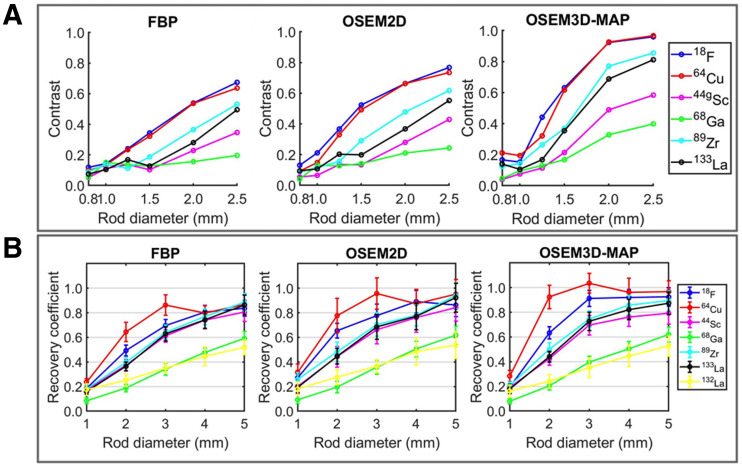
(A) Normalized contrast as function of rod size for different radionuclides in Derenzo phantom. (B) Impact of radionuclide and reconstruction method on measured recovery coefficients in NEMA image-quality phantom. ^18^F, ^64^Cu, ^44^Sc, and ^68^Ga data were taken from Ferguson et al. (*14*). 2D = 2-dimensional; 3D = 3-dimensional; FBP = filtered backprojection; MAP = maximum a posteriori; OSEM = ordered-subsets expectation maximization.

Figure 6 plots the contrast between the rods and background for each of the 6 triangular segments in the Derenzo phantom and the recovery coefficients as a function of rod size in the NEMA image-quality phantom. Additional comparisons of imaging performance metrics between radionuclides for different reconstruction algorithms are included in Supplemental Figure 5. ^133^La exhibits contrast similar to that of ^89^Zr and is superior to ^68^Ga and ^44^Sc for larger rod diameters. ^132^La was not included in the contrast comparison because of the low contrast for each rod diameter. The rods could not be distinguished below 1.25 mm in diameter for the higher-energy positron emitters ^44^Sc and ^68^Ga and 1 mm for the lower-energy positron emitters ^18^F and ^64^Cu. This blurring is due to the extrinsic scanner resolution, which is significantly impacted by the positron energy and therefore range. The recovery coefficient comparison demonstrates that ^133^La exhibits favorable performance compared with ^68^Ga and ^132^La.

### Radiolabeling

Radiolabeling was performed at 90°C for 30 min and analyzed with radio–thin-layer chromatography using 0.1 M citric acid buffer as the mobile phase. The ^133^La-DOTA, ^133^La-PSMA-I&T, and ^133^La-macropa complexes remained close to the thin-layer chromatography baseline (R_f_, 0.1–0.2), whereas unreacted ^133^La migrated toward the solvent front (R_f_, 0.9–1.0). Titration of ^133^LaCl_3_ (*n* = 3) yielded an apparent molar activity of 130 ± 15 GBq/µmol with DOTA, 73 ± 18 GBq/µmol with PSMA-I&T, and 206 ± 31 GBq/µmol with macropa.

### Preclinical PET Imaging

**FIGURE 7.**
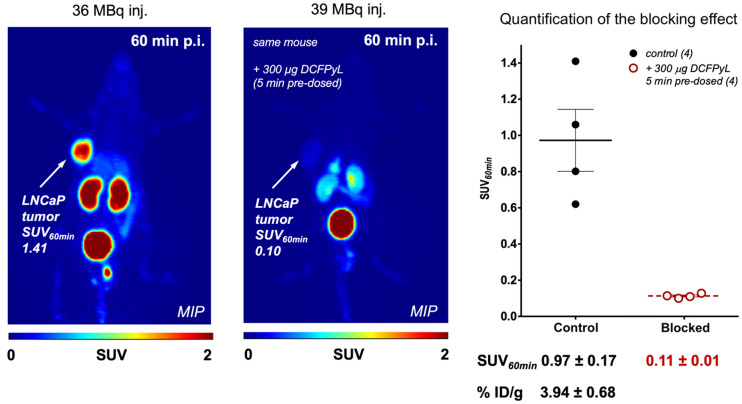
Representative PET maximum-intensity-projection images at 60 min of ^133^La-PSMA-I&T with and without predose of DCFPyL in LNCaP tumor–bearing mice. ID = injected dose; MIP = maximum-intensity projection; p.i. = after injection.

Figure 7 depicts static PET images of LNCaP tumor–bearing mice 60 min after injection of 33–50 MBq of ^133^La-PSMA-I&T (*n* = 4). Tumor uptake was significant, reaching an SUV_mean_ of 0.97 ± 0.17 after 60 min. The SUV_mean_ for muscle was 0.05 ± 0.01, resulting in a tumor-to-muscle ratio of 22.4 ± 4.5. Mice predosed with 300 µg of DCFPyL 5 min before ^133^La-PSMA-I&T injection exhibited significant tumor blocking, with a tumor SUV_mean_ of 0.11 ± 0.01 after 60 min. Most other radioactivity was excreted into the kidneys and urinary bladder.

## DISCUSSION

This study presents cyclotron production of ^133^La using natural and isotopically enriched barium target material, favorable fundamental PET phantom imaging characteristics of ^133^La, and the first (to our knowledge) in vivo preclinical PET tumor imaging using ^133^La-PSMA-I&T.

The new target assembly is well suited to the irradiation and processing of barium metal and BaCO_3_ target material. Using aluminum instead of silver target backings as used in previous studies (*3*,*11*) avoids production of long-lived ^107^Cd, ^109^Cd, and ^106m^Ag, thereby strongly reducing overall activation of the target, lowering operator exposure, and enabling rapid reuse. Using the target backing as an intrinsic degrader simplifies and enhances the available range of irradiation energies. The indium wire seal stayed 1–2 mm outside the target beam spot, avoiding activation and formation of radiotin isotopes. Sonicating used target disks in 18 MΩ·cm water allowed repeated reuse to make additional targets, with the same seal and target backing reused over 5 times.

Irradiating enriched ^135^BaCO_3_ at 23.3 MeV produced far more ^133^La than did other target materials, allowing production of clinically relevant ^133^La activities with significantly shorter irradiation times than using ^nat^Ba target material. Target separation gave a high ^133^LaCl_3_ yield in a 1-mL product volume, ready for radiolabeling.

Recovery of BaCO_3_ target material demonstrated feasibility for cost-effective recovery of expensive isotopically enriched ^135^BaCO_3_. XRD analysis of recovered BaCO_3_ showed complete conversion of the BaC_2_O_4_ intermediate and a pure recovered product, validating target material recovery and highlighting the potential for substantially improved economics with a simple and inexpensive recovery process. Radiolabeling DOTA, PSMA-I&T, and macropa with ^133^La achieved high apparent molar activities for fresh and recovered BaCO_3_ target material, similar to radiolanthanum chelation in previous studies (*3*–*5*,*16*).

Using isotopically enriched ^135^BaCO_3_ target material permits selective production of ^133^La and ^135^La compared with ^nat^Ba target material. Performing irradiations at energies of 23.3 MeV or higher significantly increases ^133^La production via the ^135^Ba(p,3n)^133^La reaction and reduces ^135^La production from the ^135^Ba(p,n)^135^La reaction, which is ideal for PET imaging applications. Irradiating at 11.9 MeV with enriched ^135^BaCO_3_ is ideal for producing large activities of pure ^135^La for AET. Using these 2 distinct reactions permits production of a variety of ^133/135^La isotopic blends on a variable-energy cyclotron.

Another production route could use isotopically enriched ^134^BaCO_3_ target material to produce ^133^La via the ^134^Ba(p,2n)^133^La reaction. This would enable ^133^La production on lower-energy cyclotrons because of the ^134^Ba(p,2n)^133^La cross-section threshold at 12 MeV as opposed to the 20 MeV threshold for the ^135^Ba(p,3n)^133^La reaction. The lower natural isotopic abundance of ^134^Ba (2.4%) than of ^135^Ba (6.6%) would result in a higher isotopic enrichment cost. However, this is a compelling option for PET centers with lower-energy cyclotrons because of the 95.4% recovery yield of BaCO_3_ target material demonstrated in this study.

PET phantom imaging clearly showed that ^133^La exhibits spatial resolution and contrast superior to those of ^44^Sc, ^68^Ga, ^132^La but similar to those of ^89^Zr. As expected, lower positron emission energy leads to improved spatial resolution (*17*) and results in superior image quality for ^133^La versus ^132^La, ^68^Ga, and ^44^Sc. This superiority is clearly translated to preclinical imaging, as evidenced by high spatial resolution. Even with the lower positron branching ratio of ^133^La (7.2%) versus other PET radionuclides (96.7% ^18^F, 88.9% ^68^Ga, and 41.2% ^132^La), the LNCaP tumor was clearly defined, reaching an SUV_mean_ of 0.97 ± 0.17 or 3.94 ± 0.68 %ID/g at 60 min after injection. For ^68^Ga-PSMA-I&T, 4.95 ± 1.47 %ID/g uptake into LNCaP tumors was reported in an ex vivo biodistribution study (*18*). As discussed previously (*3*), in vivo studies involving retention and dosing of ^133^La decay daughter ^133^Ba would be useful to address this potential limitation; however, as shown by Newton et al. (*19*), most ^133^Ba activity could be expected to be excreted within 10 d after injection.

Since lanthanum and actinium are group 3 elements with similar chemical properties, ^133^La is highlighted as a strong candidate to become a clinical PET imaging surrogate for ^225^Ac α-therapy, with PET imaging characteristics superior to those of ^132^La. As established in this study and previously (*3*), compared with ^132^La, ^133^La has superior inherent cyclotron production characteristics, a lower positron energy that translates to a higher spatial resolution, and lower-energy and lower-abundance γ-emissions that would translate to a lower patient and operator dose. These characteristics suggest that ^133^La represents an attractive candidate for diagnostic PET imaging and treatment monitoring of clinical ^225^Ac targeted α-therapy and research involving ^135^La AET.

## CONCLUSION

This work demonstrates the strong potential of ^133^La to serve as a theranostic PET imaging agent with ^225^Ac targeted α-therapy or ^135^La AET. The first preclinical in vivo PET imaging studies on LNCaP tumors resulted in high spatial resolution and contrast. Phantom imaging of ^133^La demonstrated that fundamental PET imaging properties, including spatial resolution, contrast, and recovery coefficient, were superior to those of other PET radiometals such as ^68^Ga, ^44^Sc, and ^132^La and similar to those of ^89^Zr. With cyclotron production routes capable of generating clinically relevant ^133^La activities, and with demonstrated feasibility for performing high-yield recovery of expensive isotopically enriched ^135^BaCO_3_ target material, ^133^La appears to be a promising radiometal candidate for high-resolution PET imaging as a PET/targeted α-therapy theranostic pair with ^225^Ac or a PET/AET theranostic pair with ^135^La.

## DISCLOSURE

The Dianne and Irving Kipnes Foundation supported this work. Bryce Nelson received graduate scholarship funding from Alberta Advanced Education and the Natural Sciences and Engineering Research Council of Canada. No other potential conflict of interest relevant to this article was reported.

## Notes

KEY POINTS **QUESTION:** Is the positron emitter ^133^La suitable for in vivo tumor imaging, and how do its production techniques and fundamental imaging characteristics compare with those of other PET radionuclides? **PERTINENT FINDINGS:** Phantom imaging showed the PET spatial resolution of ^133^La to be superior to that of ^68^Ga, ^44^Sc, and ^132^La and comparable to that of ^89^Zr. Preclinical imaging with ^133^La-PSMA-I&T in tumor-bearing mice clearly delineated tumors with high spatial resolution. Robust, economical, high-yield cyclotron ^133^La production was demonstrated using recoverable isotopically enriched ^135^BaCO_3_ target material. **IMPLICATIONS FOR PATIENT CARE:** This study showed that ^133^La is a strong candidate to improve patient care by providing PET imaging of tumors as a theranostic pair with ^225^Ac targeted α-therapy or potential ^135^La AET.
